# An open-source representation for 2-DE-centric proteomics and support infrastructure for data storage and analysis

**DOI:** 10.1186/1471-2105-9-4

**Published:** 2008-01-07

**Authors:** Romesh Stanislaus, John M Arthur, Balaji Rajagopalan, Rick Moerschell, Brian McGlothlen, Jonas S Almeida

**Affiliations:** 1 The University of Texas M. D. Anderson Cancer Center, 1515 Holcombe Blvd., Houston, TX 77030, USA; 2 Medical University of South Carolina, 171 Ashley Ave., Charleston, SC 29425, USA; 3 Virginia Bioinformatics Institute, Washington Street, MC 0447, Blacksburg, VA 24061, USA; 4 BioRad Laboratories, 1000 Alfred Nobel Dr., Hercules, CA 94547, USA

## Abstract

**Background:**

In spite of two-dimensional gel electrophoresis (2-DE) being an effective and widely used method to screen the proteome, its data standardization has still not matured to the level of microarray genomics data or mass spectrometry approaches. The trend toward identifying encompassing data standards has been expanding from genomics to transcriptomics, and more recently to proteomics. The relative success of genomic and transcriptomic data standardization has enabled the development of central repositories such as GenBank and Gene Expression Omnibus. An equivalent 2-DE-centric data structure would similarly have to include a balance among raw data, basic feature detection results, sufficiency in the description of the experimental context and methods, and an overall structure that facilitates a diversity of usages, from central reposition to local data representation in LIMs systems.

**Results & Conclusion:**

Achieving such a balance can only be accomplished through several iterations involving bioinformaticians, bench molecular biologists, and the manufacturers of the equipment and commercial software from which the data is primarily generated. Such an encompassing data structure is described here, developed as the mature successor to the well established and broadly used earlier version. A public repository, AGML Central, is configured with a suite of tools for the conversion from a variety of popular formats, web-based visualization, and interoperation with other tools and repositories, and is particularly mass-spectrometry oriented with I/O for annotation and data analysis.

## Background

The post genomic era has seen an increasing effort put into systematic surveys of various proteomes. Consequently, proteomics is rapidly evolving into a high throughput experimental approach that enables the identification, for example, of differentially expressed proteins as biomarkers for disease and pathogenesis. Similarly, there is a critical need for central repositories and common data formats to make the most of the copious amounts of data generated by the different screening initiatives. The higher methodological complexity of proteomics makes data integration a challenge, greatly complicated by the fact that there are no comprehensive data structures in many proteomic fields. In spite of the fact that separation by 2-dimensional gel electrophoresis (2-DE) followed by spot identification by mass spectrometry has been a major workhorse and a versatile tool in discovery proteomics [1,2], it remains under-supported by stable data formats and repositories. High resolution 2-DE provides a powerful tool for the reproducible separation, visualization, and quantification of thousands of proteins in a single gel. The increasing variety and amount of proteins being separated and the number of researchers using the 2-DE method has generated an immense diversity of datasets produced by different laboratories and using different instruments.

The lack of common formats has had an even more pernicious effect at the level of centralized data reposition, as well as in the development of incipient publicly available open source software that would enable the experimental biologist to analyze 2-DE data. The field relies on a fragmented collection of proprietary tools associated with specific instruments. A consequence of the lack of stable open formats and the piecemeal processing by instrument specific data analysis tools is that the experimental context for the generation of specific datasets is rarely stored with the raw data. Interestingly, the availability of tools for MS-based proteomics screening is far better, with two standards having emerged under the patronage of different organizations, mzXML [3] and mzData [4]. The emergence of a stable data standard, or format, coupled with a public repository, catalyzes the subsequent establishment of additional specialized or more abstract formats, as well as analysis and visualization tools (e.g., for mzXML [5] and mzData [6]). The key ingredient for this process is a consistent data format, which gives the tool creator a stable platform from which to work.

However, this is being changed by the work recently undertaken by HUPO-PSI-GEL. Specifically, the Gel Markup Language (gelML; currently in its 2^nd ^milestone; [7]) and GelInfoML (currently in its 1^st ^milestone; [8]) are hoping to fill the lack of gel standards. gelML captures a gel electrophoresis experiment from experimental procedures up to sample processing. It makes use of the Functional Genomics Experimental Object Model [9,10], which defines common components found in many biological experiments and extensions thereof according to the authors. Along with gelML, HUPO-PSI is working on guidelines for the documentation of proteomics experiments known as the minimum information about proteomics experiment, or MIAPE [11]. gelML, along with GelInfoML, which is based on MIAPE guidelines, hopes to be a comprehensive data standard for 2-D gel electrophoresis.

There is a clear need for stable open data structures, data analysis tools, and data repositories in 2-DE-centric proteomics that enable the comprehensive representation of the data from raw image pixel values to the experimental methodology used to generate it. Several groups, including ours, have recently made incremental advances toward that goal [12-14]. This report describes resources built around the annotated gel markup language (AGML) format [13], which has been improved along with converters and analysis and management tools. The public repository, known as AGML Central [15], and its conversion utilities that allow data upload in a variety of formats were correspondingly upgraded. The emphasis on interoperation and cross-reference with other open formats was also reflected by the extended support for spot identification by Mass Spectrometry, which is addressed by a number of stable, open formats.

## Results

### XML representation of 2-dimensional gel electrophoresis experiments

#### Concept

The creation of a comprehensive representation for 2-D gel electrophoresis was based on two criteria: independence of data, and an adequate minimum amount of data. Fulfillment of these criteria allows another scientist in the same field to replicate a given experiment. Thus, AGML 2.0 includes a substructure describing the protocol used in running the 2-DE experiment. This was achieved by incorporating other open data formats developed to describe gel-centric experimental protocols into AGML 1.0 [13] (see below).

Similarly, AGML 2.0 also includes a dedicated MS data substructure that provides the option of using already established MS data structures [3,12] or provides the link to a proteomics identification database such as the proteomics identifications (PRIDE) database [16].

#### AGML 2.0 data structure

**Figure 1:**
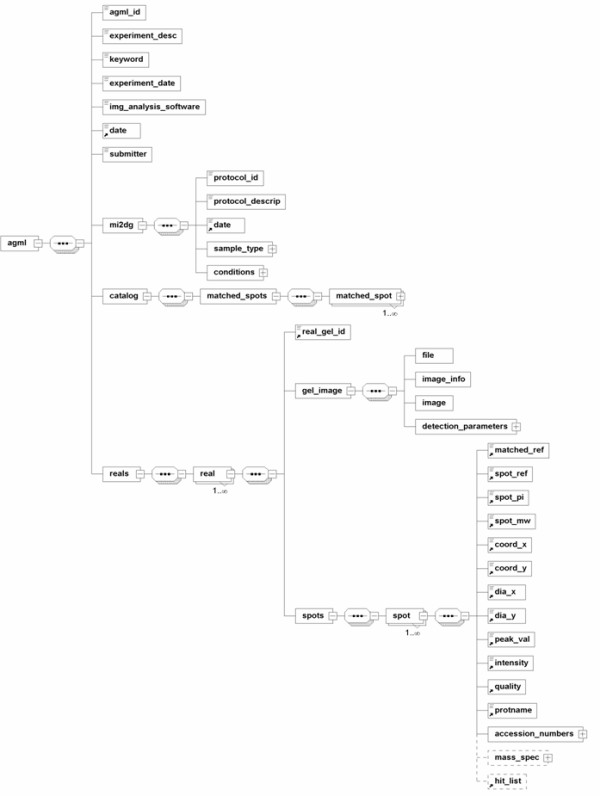
**Top level view of the AGML XML data structure**. AGML represented as a UML class diagram can be found at the project website [25]. The element 'detection_parameters' (a child of 'gel_image') enables AGML 2.0 to handle DIGE gel images. Also, the fact that AGML stores the raw gel image enables their reanalysis by other means. Please note that, for clarity, not all elements are shown in the figure.

An AGML document can be broadly divided into a) an identification section, b) a protocol section, and c) a gel section (Figure 1).

a) The identification section consists of information that identifies the experiment and the instruments used in the analysis of data. Two identifiers deserve particular notice. The *experiment_desc *element can be used to describe the experiment with keywords or a descriptive sentence. The *agml_id *element is a unique identifier generated automatically for data submitted to AGML Central.

b) The protocol section, also known as the minimum information about 2-D gel electrophoresis (MI2DG) [17], is intended to put the obtained data in context with the methodological and biological information. We have abstracted this from the spot information for several reasons. The main reason is that the sample processing method is not directly relevant to the image analysis; however, it is important in the final analysis of data. Furthermore, separating the protocol section and the spot information into different subsections allows for their independent description. Additionally, the protocol section includes a number of elements describing the experimental protocol, and provides important covariates for data analysis and semantic searching of the AGML data structure. It contains several elements that identify the sample, protocol type, and conditions used for the electrophoresis run.

In the AGML Central infrastructure, the MI2DG information has a dedicated management web portal so that protocols can be created and modified based on an existing protocol. This eliminates the need for the researcher to repeatedly key in all of the information, and allows researchers to easily modify a protocol based on previously published protocols. An additional advantage of the autonomous protocol database [17] is that researchers have archived catalogs of all of the protocols used in their labs. MI2DG entries have their own unique identifiers (*mi2dg_id*) and can be independently referenced.

c) The gel information section consists of spot information divided into two sections: *catalog *and *reals*. The *catalog *section describes the aligned spots in all of the real gels. The subelement *protname *describes the most likely protein ID out of the possible candidates described in the *hit_list*. The *reals *element describes an individual gel (real) that comprises one or many spots. Each *spot *element describes an individual spot in a gel by defining many subelements that, taken together, uniquely identify the spot. The *matched_ref *element identifies a catalogued gel and is therefore present in both the *catalog *and *real *sections. On the other hand, *spot_ref *describes an ID given by the acquisition system (e.g., PDQUEST identifies spots with *ssp *string). This enables linkage of the data stored in the acquiring machine to AGML for auditing purposes.

The *gel_image *element contains all of the details of the image file uploaded by the user. The *image *subelement contains the whole image file as base64-encoded binary data. This element can store the raw image as well as the processed image. Additionally, the *file *and *image_info *elements contain image specific information that is useful in further analysis of the image. The *detection_parameters *element and its subelements in the *gel_image *section enable the inclusion of images scanned at different wavelengths, such as Differential In-Gel Electrophoresis (DIGE) gel images, into AGML. This additional tag gives AGML the ability to store any 2-DE gel, regardless of the number of different wavelengths used in the analysis. Inclusion of the gel images results in the document being very large; however, having the image in the document provides the user with immediate access to the original data.

**Figure 2:**
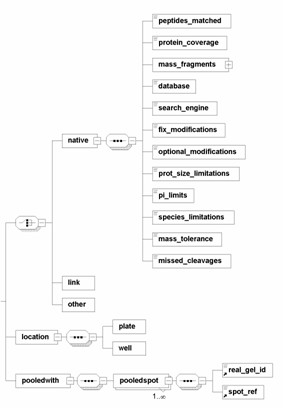
**View of the sub element <mass_spec> structure**. The non-compulsory 'mass_spec' provides the option to a) store the mass spec data in a native format provided by AGML, b) store it in another format such as mzXML or mzData, or c) add a link to a location where the data is stored, such as PRIDE. Additionally, the elements 'location' and 'pooledwith' (children of 'mass_spec') can capture the location of the plate well where the spot was deposited or pooled respectively.

AGML version 1.0 [13] provided elements to include mass-to-charge and intensity pairs to describe the mass spectrometry results obtained for individual 2-DE spots. Version 2.0 extends this by giving different options to store the mass-spectrometry data, which underscores the fact that 2-DE experiments are tightly coupled to the mass spectrometric identification (Figure 2). This element acts only as a place holder for connecting established mass spectrometry-centric XML data structures such as mzXML [3] and mzData [12], and for describing mass spectrometric information for the respective spot. Extension to accommodate other mass spectrometric schemas could easily be achieved by referencing those using standard XML namespace rules [18]. Additionally, using the 'link' element, one can identify whether the proteomic data has also been submitted or identified and placed in a repository such as PRIDE [16]. The support for 2-DE and MS experimental designs is also extended to the manipulation of the gels, a specific requirement to accommodate AGML-centric laboratory management systems. Additional elements under *mass_spec *can also identify the location (*location*) on the plate used for mass spectrometry analysis where the sample was applied. The element *pooledwith *can identify whether the sample spotted on the plate came from a pooled sample of spots from many gels.

#### AGML Central: data repository and analysis framework

**Figure 3:**
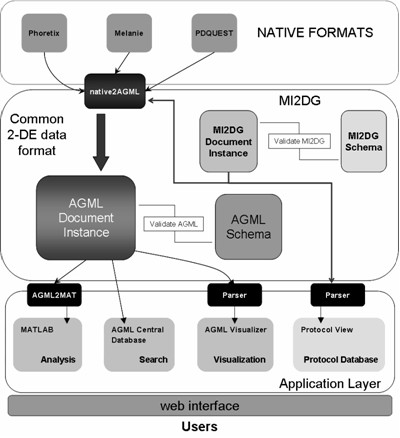
**AGML Central web infrastructure**. AGML XML format describing a 2-DE experiment is central to the AGML Central architecture. The web infrastructure is written in PHP programming language, a widely-used general-purpose scripting language, and the storage of the XML instance documents is provided by PostgreSQL, an open source object-relational database management system. The AGML document is stored as a logical unit within the database. This eliminates the need to store the document as blobs and also provides for fast retrieval of the data. Analysis software is written in MATLAB^® ^(The MathWorks, Inc.), a technical computing environment ideal for handling high dimensional data.

AGML Central [15], a web-based analysis pipeline created around the AGML format, was expanded into a 2-DE data warehouse (Figure 3). The first set of programs developed were converters that would translate native data files into AGML data files. Currently, there are converters for PDQUEST (Bio-Rad, CA, USA), Phoretix2D (Non-linear Dynamics), Melanie (GenBio SA), and DeCyder DIGE (GE Healthcare). The software programs described below were developed based on the AGML structure. Thus, there is no need to re-write these applications to work with individual files generated by different analytical instruments as long as they are in AGML format.

AGML Central includes AGML Visualizer, an instrument-neutral Java applet that visualizes 2-DE gels. Built-in capabilities such as searching, displaying the experimental protocol, and displaying individual spot information makes the analysis of 2-DE data easy. This feature also greatly enhances the dissemination of the 2-DE data by allowing the data generated from one instrument to be viewed, even in the absence of the corresponding acquisition instrument. AGML Visualizer can be launched in two different ways: as an applet, or through the Java Web Start infrastructure. Using the Java Web Start infrastructure, an AGML document can be opened on a local hard drive. However, the applet makes use of the AGML file that has been deposited into the AGML Central database.

**Figure 4:**
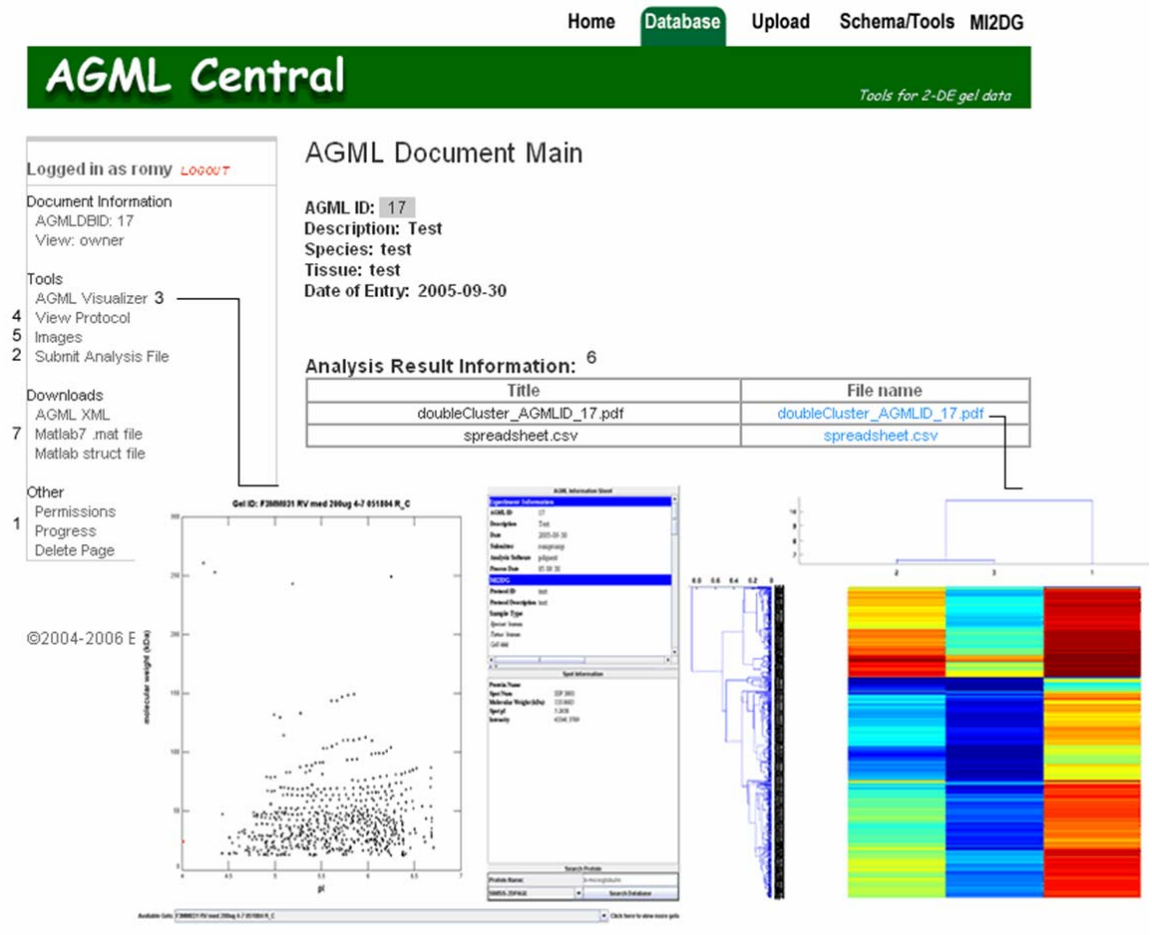
**AGML Central web site displaying the AGML Document Main page**. This page gives access to all of the information relating to a 2-DE experiment. The owner of the page can also give permissions to others to view the experiment, check progress, and delete the submitted files (1). Collaborators of the project can also submit files to the project (2) or view the experiment using AGML Visualizer (3). They can also view the 2-DE protocol used for the experiment by clicking on the view protocol link (4). Raw images can be viewed or downloaded by going to the images link (5). All of the project files are described on this page under analysis result information (6) and can be viewed or downloaded. Additionally, the AGML XML file and MATLAB^® ^mat files are available for download from this page for the experiment (7). Thus the MATLAB^® ^code written for this project can be used to analyze the 2-DE data directly without further manipulation.

Another advantage of using the AGML Central framework is the ability to make use of the tools developed for the analysis of AGML files. A number of AGML-centric data analysis tools were also developed, not just to add to the functionality of AGML Central, but also to illustrate how researchers can test novel algorithms directly on database entries. This not only illustrates the use of AGML Central as a data service, but also underscores the importance of such a service to 2-DE-centric research. For example, the tool illustrated in Figure 4 provides the sort of double clustered heat map functionality that is often used to explore microarray data.

The prototypic statistical analysis applications that use AGML Central as a data service were written in MATLAB^® ^(The MathWorks, Inc., Natick, MA, USA) and are available at the AGML Central website [19]. The statistical tools currently available are for cluster analysis, principal component analysis, and normalization of 2-DE results [19].

## Discussion

AGML was developed through close interaction with bioinformaticians and experimentalists to create a common data format that is open, accessible, and encompassing of all aspects of 2-DE experiments [13]. The AGML format accommodates a description that spans from the start of the experiment to the final identification step, and as a consequence, all of the data is placed within the experimental context. The resulting definition, AGML 2.0, allows users to establish both the provenance and relevance of a 2-DE experiment, thereby enabling the development of effective search and analysis tools.

Specialized databases exist throughout the world that focus on 2-DE data [20-22], with SWISS-2DPAGE being a major database [23]. Other research efforts have been directed toward comprehensive representation of proteomics experiments, such as PEDRo [14] and HUP-ML [24]. AGML was developed as a pragmatic representation of the 2-DE-centric subdomain and can be used to interoperate with those much larger and more encompassing representations.

In spite of being the workhorse in proteomic study, a gel-centric approach has had weak bioinformatics support due to the lack of stable gel-centric data standards and formats. In order to assist with tool development and data dissemination, a public database of AGML formatted entries, AGML Central [25], was developed. This web interface comes with a visualization plug-in and a portal for retrieval and submission by external applications. For example, MATLAB^® ^(The MathWorks, Inc., Natick, MA, USA) GUI functions are available in the tools page for direct access to data to and from AGML Central. The ability to map AGML XML format to a MATLAB^® ^'struct' (agml) enables statisticians and bioinformaticians to create algorithms based on it. This MATLAB^® ^struct can hold information extracted from an AGML XML document; hence, users of this format can, by extension, use any algorithm developed for the struct 'agml'. This feature allows the development of AGML Central-based pipelines and analysis tools. Additionally, the results of the analysis using other methods can also be submitted to AGML Central, to be appended to the corresponding entry. Although AGML Central is a public repository, all data submitted is private by default. The owner of the data can decide to make it public by providing selective access. There are currently 26 entries, of which only 2 have been made public. However, 14 of the entries have been designated for collaboration. It is our hope that as collaboration is completed all data will be made public.

The AGML concept and its implementation facilitate the management of proteomic data coming from diverse labs using different instruments and protocols, and enable the creation of much needed public 2-DE databases [26]. For this reason, the AGML format provides a wider community of developers (through the accompanying open source project) and a larger audience of users (such as bioinformaticians and statisticians) with a way to access information generated by 2-DE experiments, thus enabling them to develop comprehensive data mining algorithms that allow for exploratory and confirmatory data analysis. For example, Oates et al [27] used the AGML Central infrastructure to manage, integrate, and analyze 2-DE data to identify biomarkers that differentiate the two most common causes of acute renal failure. They used AGML Central to disseminate both their protocol and proteomic data in the AGML format to their bioinformatics collaborators. They then used the AGML data structure in the MATLAB^® ^native format, which is provided by AGML Central, to do exploratory analysis on their proteomic data. Using AGML Central allowed the collaborators access to all of the information at any time, thus streamlining their collaborative effort to get results faster. Additionally, a nascent controlled vocabulary exists for AGML; please see Minimum Ontology for 2DE Gel Electrophoresis [28] for more information on this effort. Completion of this work will give the AGML format true agility and the ability to work with semantic web technologies.

The standards being created by HUPO-PSI-GEL for 2-D gel electrophoresis data markup, gelML and GelInfoML [7] and two analogous MIAPE modules (GE and GI; [29]), hope to encompass more details and be a comprehensive data standard for 2-D gel electrophoresis as a whole. gelML and GelInfoML are both based on the Functional Genomics Experiment [9] modeling framework. While these efforts will ultimately result in community-based data standards, AGML was created to answer this need more pragmatically. Since its inception in 2004 [13], AGML has been more interested in getting the data to tool makers. In the process AGML has acquired many of the features that are being proposed. Briefly, the <mi2dg> elements can be analogous to GelInfoML, and the <reals> can be analogous to gelML. Once stable gelML and GelInfoML standards are published, AGML documents will be made available to be translated to these standards, thereby making the data available for any tools developed for HUPO-PSI-GEL standards.

Overcoming barriers in data flow is a central theme in the route toward Systems Biology and this is especially true for rapidly expanding methodologies such as those developed for proteomics research. Rapid growth of the field has seen the emergence of high throughput instruments from different vendors that use many different proprietary data standards that, due to the lack of data interoperability, limit data integration. This fact is underscored by the formation of the Interoperable Informatics Infrastructure Consortium, whose major goal is to eliminate barriers to application interoperability, data integration, and eventually knowledge sharing [30]. Additionally, work undertaken by HUPO-PSI to advance the field of proteomics data standards also points to the need that exists in the area of data interoperability in proteomics [31].

## Conclusion

The gel-centric AGML data structure is a comprehensive format for the representation of 2-DE proteomic data. It seeks to address the glaring need for a pragmatic format in which both experimental results and their experimental context can be represented. The future of 2-D electrophoresis tool development may depend on stable standards and formats being devised and used. The stability that comes with such endeavors is critical to enabling the development of open source data analysis tools that are long overdue for gel-centric proteomics.

## Methods

Python, Matlab, PHP and bash Programming languages were used in the development of this project. UML diagrams were used to visualize the data structure and XML was used in creating the AGML data structure.

## Authors' contributions

RS and JSA conceived and wrote the manuscript. RS designed and implemented the object model. JAM, BR, RM and BM provided experimental expertise. All authors read and approved the final manuscript.
